# www.jisponline.com

**DOI:** 10.4103/0972-124X.71779

**Published:** 2010

**Authors:** Neha Gupta

**Affiliations:** *Project Editor, Journal of Indian Society of Periodontology, H 11 A South Avenue, Thiruvanmiyur, Chennai - 600 041, India. E-mail: nehagupta.jisp@gmail.com*

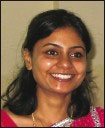



I cannot think of any other title that would be concise and at the same time express the huge magnitude of the change that has helped in planting ISP firmly on the world periodontists’ map. The dynamic thinking of the editor, my mentor, Dr D Arunachalam has given *Journal of Indian Society of Periodontology (JISP)* a makeover and helped it carve a niche in the mind of the dental scientific community.

This new avatar, our editor’s brainchild, eliminated geographical boundaries by enabling electronic submission and review of all articles. The regular publication with international participation and registration with DOI have made *JISP* one of the first Indian journals on dentistry to be indexed with Pubmed, archived with PMC and have a regular citation tracking.

Keeping pace with the expanding web usage, *JISP* now has its own blog, and a Wikipedia page. It is also available as an e-book and in mobile optimised formats and has its records added to library search engines (Discover Solutions) and secondary aggregation agencies like ProQuest.

I am grateful to the periodontal fraternity for selecting us to showcase their original research and to our expert review panel in maintaining the highest quality of scientific content. It is indeed an honour to be associated with this new chapter of *JISP*, www.jisponline.com

